# β^3^-tripeptides act as sticky ends to self-assemble into a bioscaffold

**DOI:** 10.1063/1.5020105

**Published:** 2018-05-01

**Authors:** Mark P. Del Borgo, Ketav Kulkarni, Mary A. Tonta, Jessie L. Ratcliffe, Rania Seoudi, Adam I. Mechler, Patrick Perlmutter, Helena C. Parkington, Marie-Isabel Aguilar

**Affiliations:** 1 Department of Biochemistry and Molecular Biology, Monash University, Clayton, VIC 3800, Australia; 2 Biomedicine Discovery Institute, Monash University, Clayton, VIC 3800, Australia; 3 Department of Physiology, Monash University, Clayton, VIC 3800, Australia; 4 Department of Chemistry and Physics, La Trobe Institute for Molecular Science, La Trobe University, Bundoora, VIC 3086, Australia; 5 School of Chemistry, Monash University, Clayton, VIC 3800, Australia

## Abstract

Peptides comprised entirely of β^3^-amino acids, commonly referred to as β-foldamers, have been shown to self-assemble into a range of materials. Previously, β-foldamers have been functionalised via various side chain chemistries to introduce function to these materials without perturbation of the self-assembly motif. Here, we show that insertion of both rigid and flexible molecules into the backbone structure of the β-foldamer did not disturb the self-assembly, provided that the molecule is positioned between two β^3^-tripeptides. These hybrid β^3^-peptide flanked molecules self-assembled into a range of structures. α-Arginlyglycylaspartic acid (RGD), a commonly used cell attachment motif derived from fibronectin in the extracellular matrix, was incorporated into the peptide sequence in order to form a biomimetic scaffold that would support neuronal cell growth. The RGD-containing sequence formed the desired mesh-like scaffold but did not encourage neuronal growth, possibly due to over-stimulation with RGD. Mixing the RGD peptide with a β-foldamer without the RGD sequence produced a well-defined scaffold that successfully encouraged the growth of neurons and enabled neuronal electrical functionality. These results indicate that β^3^-tripeptides can form distinct self-assembly units separated by a linker and can form fibrous assemblies. The linkers within the peptide sequence can be composed of a bioactive α-peptide and tuned to provide a biocompatible scaffold.

## INTRODUCTION

The assembly of bioscaffolds using a bottom-up approach offers significant advantages over naturally derived polymer structures, in terms of greater design flexibility. Peptides, in particular, can be designed and synthesised to adopt secondary structures, typically β-sheets and α-helices, which then self-assemble to give higher order structures.^1–3^ Peptide-based self-assembling systems have been used by a number of groups to create a variety of materials for tissue engineering and biomedicine.^3–10^ The main advantages of using peptide self-assembly include the ability to decorate with functional groups at the monomer level and the inherent biocompatibility. However, peptides are prone to proteolytic degradation with consequent alterations in the secondary structures which provide the basis for self-assembly. The resulting alteration of sequence or environmental conditions can lead to the loss of the critical self-assembly motif.

We have recently reported the self-assembly of peptides comprised entirely of β^3^-amino acids.^11–13^ These peptides possess an extra methylene group in their backbone and are commonly referred to as β-peptide foldamers.^14–17^ Peptides containing acyclic β^3^-amino acids form a unique 14-helical structure that is not adopted by α-peptides, which, in combination with N-terminal acetylation, creates a self-assembly motif. The 14-helix is of particular interest as it has almost three amino acids per turn, which creates three aligned faces of the helix. These peptides are also resistant to protease degradation^18–20^ and, in contrast to α-peptide-based materials, the unique self-assembly is sequence independent, which offers the ability to tailor the sequence towards a specific function. Most notably, the crystal structures of a β^3^-hexapeptide and a β^3^-tripeptide demonstrate that these backbone structures are superimposable, and therefore, an *N-*acetylated β^3^-tripeptide is all that is required for self-assembly. We have created a number of materials with varying morphologies, which has led to the development of hydrogels and bioscaffolds.^21–23^ This was achieved by the attachment of pendants onto the side chain of β^3^-tripeptide foldamers. Despite the incorporation of large functional pendants that are larger than the β^3^-tripeptide template itself, there was no loss of self-assembly.

**FIG. 1.**
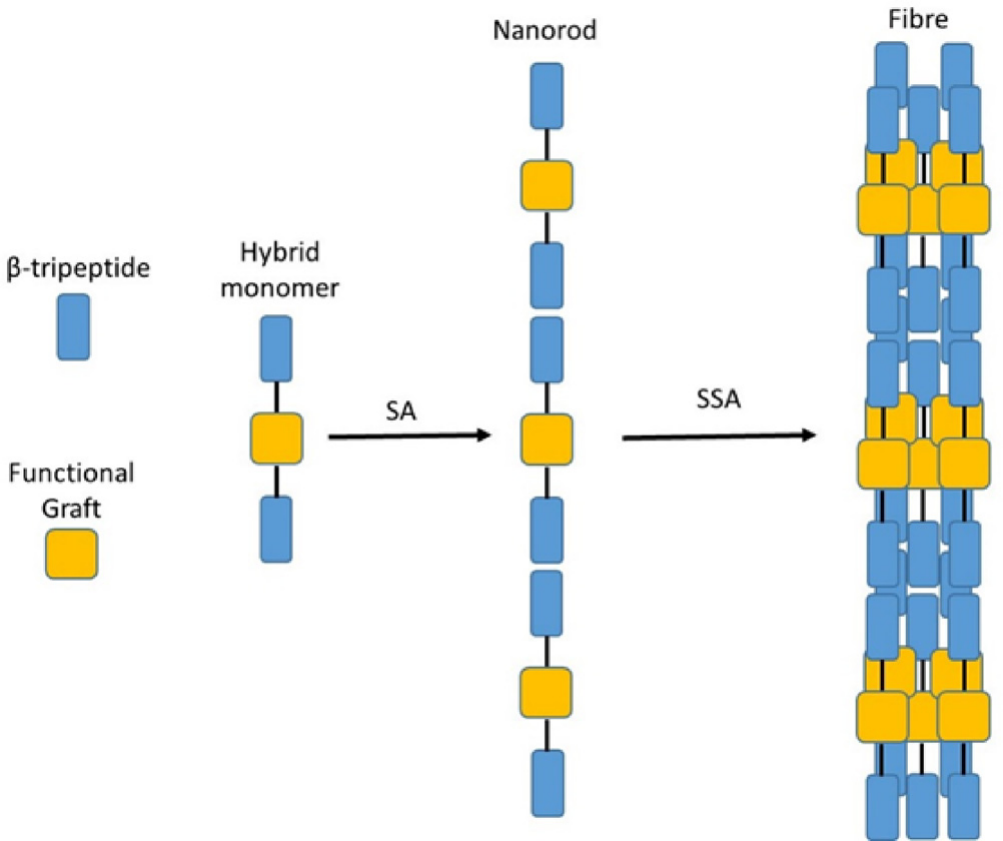
Schematic diagram illustrating the incorporation of functional groups within a b-peptide sequence (SA = self-assembly; SSA = supramolecular self-assembly).

In this study, we sought to explore and expand the self-assembly properties of *N-*acetylated β^3^-tripeptides by utilizing the β^3^-tripeptide template to initiate the self-assembly of molecules which typically do not self-assemble. We hypothesized that the presence of an N-acetylated β^3^-tripeptide sequence at each end of a non-assembling molecule (insert) is sufficient to drive self-assembly, thereby acting as “sticky-ends” (see Fig. 1). First, we investigated the structural requirements via the insertion of two different molecular linkers: (1) a flexible linker and (2) a rigid linker flanked by β^3^-tripeptides. Finally, we incorporated an α-peptide sequence, α-arginylglycylaspartic acid (RGD), with fibronectin-like activity as a larger functional insert to create a new bioscaffold for neuronal cell culture.

## RESULTS AND DISCUSSION

The goal of this study was to determine whether a β-hexapeptide, which is known to form two turns of a 14-helix and self-assemble in an axial head-to-tail manner, can be separated into two β-tripeptides and retain the ability to self-assemble. The 4 peptides used in this study were synthesised by solid phase synthesis and are detailed in Table I. Peptides **1** and **2** were initially designed to determine whether an interruption in the 14-helical structure of the β^3^-hexapeptide would compromise peptide self-assembly.

**TABLE I.** List of synthesised hybrid β-peptides.

Peptide	N-terminal β-peptide	Insert	C-terminal β-peptide	Purpose
**1**	Ac-WKL	*p*-aminobenzoic acid	WEL-COOH	Rigid linker
**2**	Ac-WKL	Aminohexanoic acid	WEL-COOH	Flexible linker
**3**	Ac-SVA	α-RGD	SVA-COOH	Biolinker
**4**	Ac-LIA	…	…	Adjust signal

**FIG. 2.**
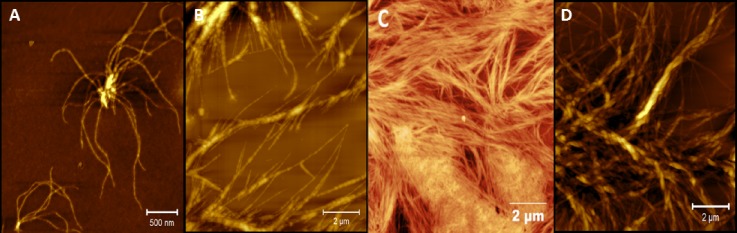
AFM images of peptides (a) **1**, (b) **2**, (c) **3**, and (d) a mixture of **3** and **4** (1:3).

Atomic force microscopy (AFM) analysis of peptides **1** and **2** demonstrated fibre formation [Figs. 2(a) and 2(b)] at similar concentrations seen in previous studies of β^3^-peptide self-assembly by our group.^11–13^ Despite the incorporation of a rigid *p*-aminobenzoic acid linker between the two β^3^-tripeptides, self-assembly was maintained in peptide **1**. AFM analysis showed the presence of aggregates, from which a number of fibres grow into an architecture with relatively short fibres. In contrast, peptide **2** displayed extensive fibre formation similar to the architectures described previously for β^3^-peptides.^11–13,22^ The morphology evident from AFM demonstrates that a flexible linker between the β-tripeptides does not hinder self-assembly and leads to longer fibres with greater coverage of the mica surface compared to **1**.

Peptide **3** was designed to include the fibronectin motif, RGD, which was inserted between two β^3^-tripeptides, in order to create a novel bioscaffold. On this occasion, we chose β^3^-amino acid residues bearing sidechains with less bulk to ensure the accessibility of the RGD sequence and hence preservation of fibronectin-like activity. AFM analysis of **3** shows a layer of interwoven fibres with a near-uniform diameter that covers the entire surface of the mica [Fig. 2(c)]. Fibres were arranged into bunches and formed a dense matt that could potentially serve as a 2-dimensional scaffold for the adherence and growth of cells. We also attempted to synthesise a range of other peptides with longer sequences including IKVAV and the anti-microbial peptide Aurein. However, rather unexpectedly, the syntheses of these molecules failed as the N-terminal β-tripeptide portion could not be attached via traditional solid phase strategies.

We have previously demonstrated that β^3^-peptide side chains can be decorated with α-peptide motifs as pendants^22^ without any perturbation of the self-assembly and these fibres translate into bioactive scaffolds and gels.^21–23^ An alternative strategy is to incorporate the functional payload, in this case the RGD motif, within the peptide backbone. Although this is a similar strategy to that of Fmoc-FRGDF and other examples where the functional motif is introduced within the sequence,^24,25^ the introduction of RGD and other epitopes within the Fmoc self-assembling peptide sequence would not be expected to affect self-assembly as the Fmoc group is responsible for self-assembly. There are also other notable examples where a functional motif is introduced between two β-sheet forming α-peptides. These peptides also do not undergo any perturbation to the self-assembled assemblies as the H-bonding networks forming the β-sheet are not compromised and yield novel optoelectronic peptide-based materials.^26–28^ In contrast, it is the H-bonding network contained within the backbone of the β^3^-peptide that drives the helical self-assembly and disruption of this structure, by introducing a payload, was predicted to inhibit this assembly.

The 14-helical structure of β^3^-tri- and hexapeptides has been shown to be crucial for the H-bonding required for self-assembly. We therefore sought to investigate the strength of this 3-point H-bonded self-assembly motif and whether it is compromised by the insertion of a rigid or flexible linker to ultimately create a hybrid α/β peptide scaffold. The results in Fig. 1 clearly showed that incorporation of such payloads, in particular flexible linkers, does not perturb self-assembly, demonstrating the resilience of the 3-point H-bonded self-assembly motif. The use of complementary repeating units, or so-called sticky ends, to promote peptide self-assembly has been shown for a number of different peptide systems. For example, flanking ion-pairs between complementary 28-residue peptides with an extended coiled coil structure were shown to promote self-assembly.^29^ This led to the design of α-helical peptides with repeating ion-pairs that self-assembled into fibres.^30^ Salt-bridges and metal triggers have also been used to promote fibrillogenesis in collagen mimetic peptides.^8,31–33^ However, these examples of complementary “sticky ends” required multiple peptides with specific amino acid sequences. However, in the present study, the flanking β^3^-peptides were composed of three β^3^-amino acids without any specific requirements in terms of the amino acid sequence.

**FIG. 3.**
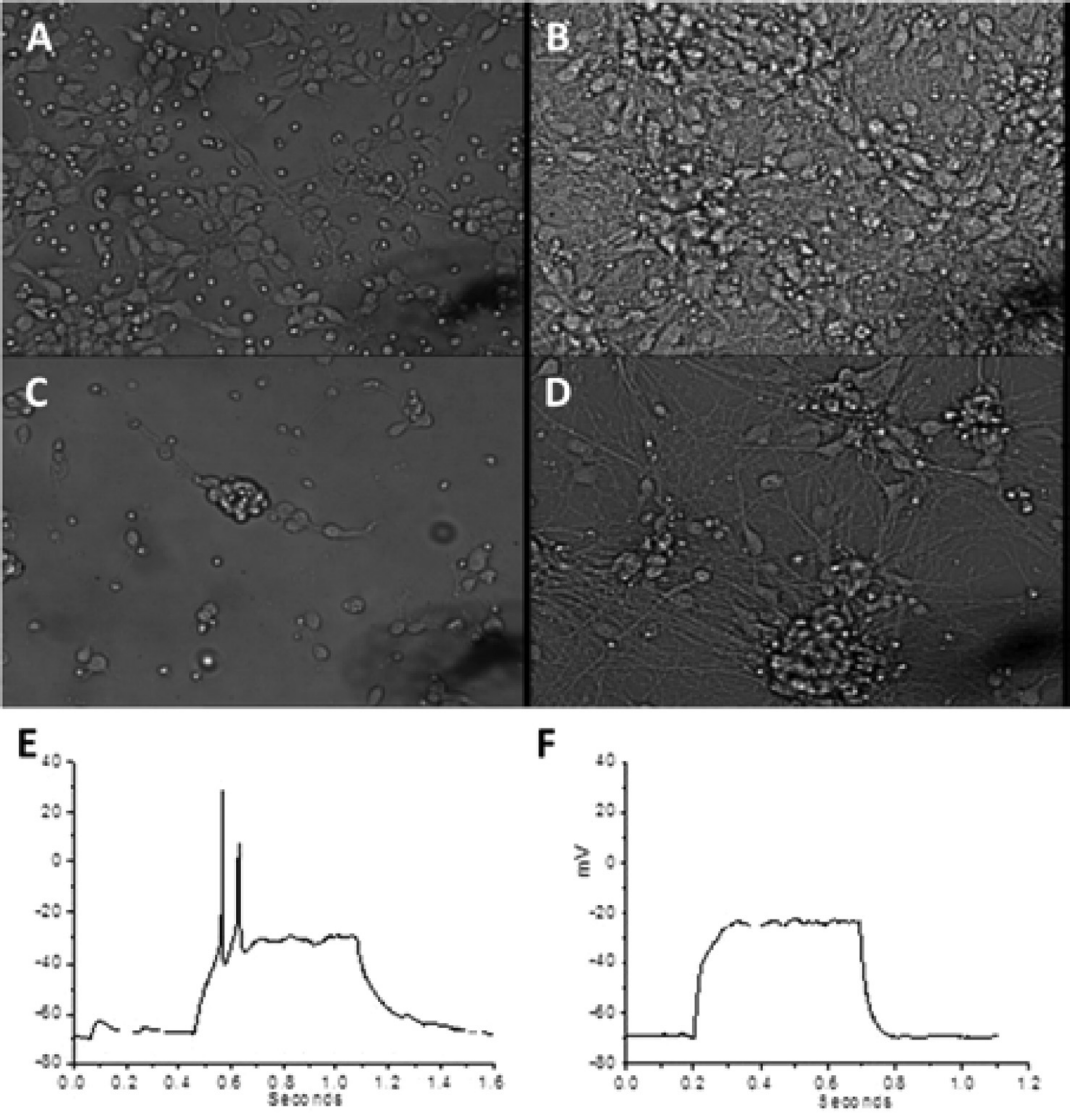
Bright field images showing primary hippocampal cell growth on PLO control slides after (a) 1 and (b) 5 days. Bright field images of neurons cultured on peptide **3** scaffold after (c) 1 and (d) 5 days. Robust resting membrane potential (RMP) and action potentials in response to a depolarizing current step in neurons grown on (e) control PLO substrate or (f) a peptide **3** scaffold.

In order to assess whether the RGD motif was accessible to cells, we coated glass slides with a scaffold composed of peptide **3** and cultured primary rat hippocampal neurons. Cells grown on the poly-l-ornithine (PLO) control appeared as expected after 1 day. Following 5 days, the cells had an extended neurite network and were proliferating and healthy [Figs. 3(a) and 3(b)]. Peptide **3** was also incubated in serum to assess possible degradation by proteases *in vitro*; however, no degradation products were observed after 1 week (Fig. S1). Cells grown on peptide **3** scaffolds were clumped on day 1, indicating that they were stressed [Fig. 3(c)], and while they were still clumped on day 5, the formation of intercellular projections was evident [Fig. 3(d)]. This indicated that scaffolds made from peptide **3** entirely were not optimal for cell growth but permitted some network formation.

Cell functions were tested by electrophysiology of primary neurons on days 5–7 in culture. Cells grown on control PLO had robust resting membrane potentials (RMPs), sodium currents, and action potentials (APs) [Fig. 3(e)]. Neurons grown on peptide **3** scaffolds had good RMP, but no sodium current and therefore neurons were unable to produce action potentials at rest [Fig. 3(f)].

β^3^-tripeptide scaffolds and other materials decorated with RGD have previously been observed to elicit cell toxicity if the concentration of RGD is too high.^34^ We hypothesised that a combination of peptide **3** and peptide **4**, a tripeptide that has previously been used by our laboratory to modulate the RGD signal,^21^ would create a co-assembled scaffold with a tunable function. In this study, we used 25 mol. % peptide **3** with 75 mol. % peptide **4**. AFM analysis of the combination scaffold showed a unique morphology [Fig. 2(d)] with some structure reminiscent of the structures seen previously with peptide **4** with some similarity to the morphology produced by the self-assembly of **3** [Fig. 2(c)].

**FIG. 4.**
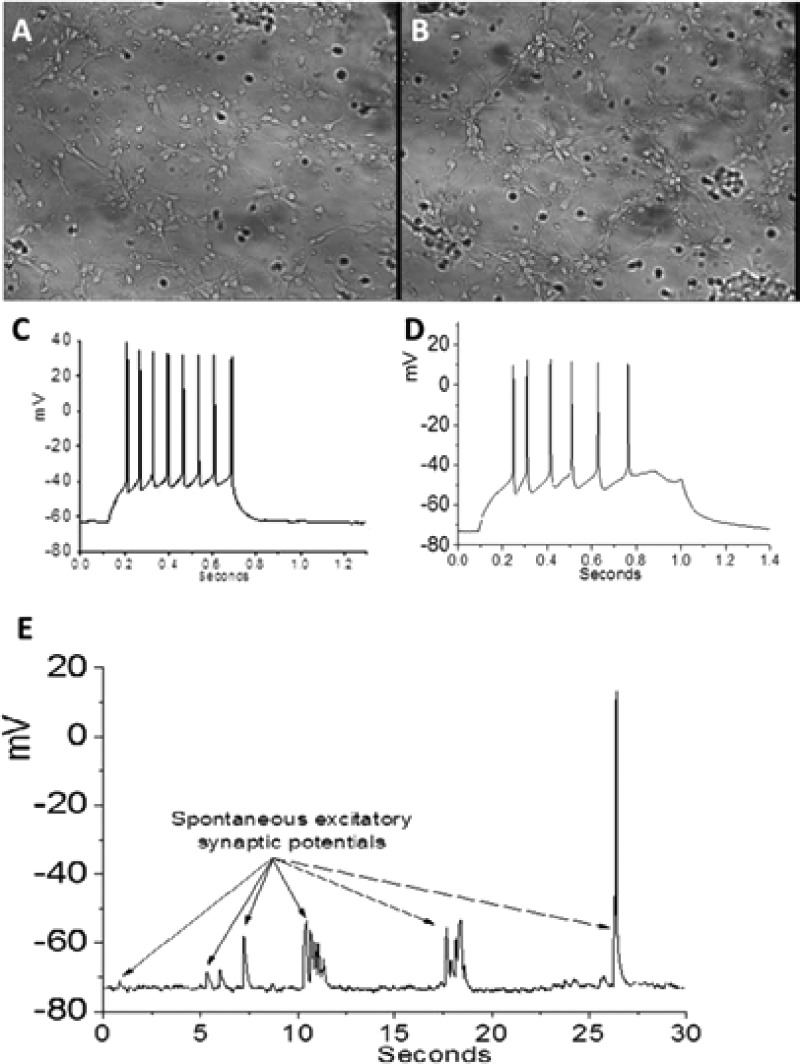
(a) Bright-field image of neuronal cell culture on PLO control plate. (b) Neurons growing 25% RGD scaffold. (c) Action potentials evoked in a neuron growing on PLO control. (d) Action potentials evoked in a neuron growing on 25% RGD scaffold. (e) Spontaneous excitatory synaptic potentials recorded for neurons growing on the 25% RGD scaffold.

Primary neurons were cultured on glass slides treated with either PLO control or with peptide **3** and **4** scaffold mixture (1:3). Following 5 days in culture, cells on PLO control and on the combination scaffold appeared equally healthy [Figs. 4(a) and 4(b)]. Patch clamp electrophysiology showed typical RMP [Fig. 4(c)] and action potentials on both substrates [Figs. 4(c) and 4(d)]. Cells grown on the combination scaffold had lower current amplitudes than cells on PLO control (*p* = 0.02). The sodium currents, and hence the action potentials, on the β^3^-peptide scaffold were not as robust as those of neurons on PLO but showed significant improvement over those grown on 100% RGD scaffolds. Excitatory post-synaptic potentials (EPSPs) occurred spontaneously in neurons on PLO (data not shown) and on the 25% RGD scaffold [Fig. 4(e)]. These spontaneous synaptic potentials gave rise to action potentials [Fig. 4(e)], indicating that the neurons are healthy enough to form functioning networks and to communicate with each other as they do in the normal brain. This functionality is the crucial indicator of neuron health and the ability of the scaffolds to support neuron survival and proliferation.

## CONCLUSIONS

We have previously shown that self-assembling β^3^-peptides can be decorated with a functional moiety through side chains modifications. Here, we have shown that a second approach to incorporate functions into these fibres has been successful. The purpose of peptides 1 and 2 was to act as a proof of principle that either a flexible or rigid linker can be used to create two distinct self-assembly units without loss of self-assembly. Once these peptides were proven to self-assemble, peptide 3 was designed and synthesised to assess whether the linker used can also possess a biological function. β^3^-tripeptides therefore act as sticky ends during self-assembly of α/β hybrid peptides to produce fibrous structures which exhibit biological activity that can be modulated by the co-assembly with non-functional β^3^-peptides. The ability of β^3^-tripeptides to self-assemble irrespective of amino acid sequence, or in this case interruption of the sequence, is remarkable and presents another avenue for the generation of new biomaterials.

## METHODS

### Synthesis and purification

Peptides were synthesised on a 0.2 mmol scale using standard Fmoc chemistry on Wang resin (0.1 mmol/g loading). The resin was swollen in Dimethylformamide (DMF) (4 ml), then soaked in Fmoc-protected β amino acid (2.1 eq. to resin loading), and dissolved in DMF (4 ml) along with N,N,N′,N′-Tetramethyl-O-(1H-benzotriazol-1-yl)uronium hexafluorophosphate (HBTU) (2 eq. to resin loading), 1-Hydroxybenzotriazole (HOBt) (2 eq. to resin loading), Dimethylaminopyridine (DMAP) (10 mol. %), and Diisopropylethylamine (DIPEA) (3 eq. to resin loading), overnight with gentle agitation. The resin was thoroughly washed with DMF (3 × 4 ml), and the Fmoc protecting group on the amino acid was removed by soaking the resin twice in 20% piperidine, with 0.1 M HOBt, in DMF (4 ml) for 15 min each. The resin was washed with DMF (3 × 5 ml), soaked in Fmoc-protected amino acid (2.1 eq. to resin loading), and dissolved in DMF (4 ml) along with HBTU (2 eq. to resin loading), HOBt (2 eq. to resin loading), and DIPEA (3 eq. to resin loading), for 2 h. The peptide elongation cycle was then repeated until the sequence was complete. After removing the terminal Fmoc protecting group on the peptide, the resin was treated with a solution of 10% v/v acetic anhydride and 2.5% v/v DIPEA in DMF (4 ml) for 30 min to afford an acetyl-capped N-terminus. The resin was washed with DMF (2 × 4 ml), CH_2_Cl_2_ (2 × 4 ml), and Et_2_O (2 × 4 ml), air dried for 10 min, and transferred to a 50 ml vial for cleavage.

Cleavage was performed on the resin by treating the resin with a cleavage solution (20 ml) comprising H_2_O (2.5% v/v), triisopropylsilane (2.5% v/v), and, in the case of peptide 3, ethanedithiol (0.5% v/v) in CF_3_COOH, for 3 h. CF_3_COOH (TFA) was then evaporated under a stream of N_2_, and the peptide was precipitated by the addition of Et_2_O (50 ml). The precipitate was filtered and redissolved in 50% aqueous CH_3_CN for lyophilisation. The peptide was redissolved in 50% aqueous CH_3_CN (5 ml), purified by injecting the sample onto a reverse-phase preparative column, and eluted over a 60 min gradient from 10% to 70% solvent B (solvent A: 0.1% TFA/H_2_O; solvent B: 0.1% TFA/CH_3_CN) with a flow rate of 6 ml/min. The fractions were collected, analysed for purity by injecting the samples onto a reverse-phase analytical column, and eluted over a 45 min gradient from 0% to 75% solvent B (solvent A: 0.1% TFA/H_2_O; solvent B: 0.1% TFA/CH_3_CN) with a flow rate of 1 ml/min.

### AFM

Atomic force microscopy (AFM) was performed on a Nanoscope IV AFM with a Multimode head (Veeco, Santa Barbara, CA, USA) using a vertical engage “E” scanner. 2 μl of peptide solution in water (0.25 mg/ml^−1^) was placed on a clean mica surface. The sample was incubated under a petri dish for 30 min and then dried with gentle stream of N_2_ gas. Images were obtained in tapping mode in air with NSC-15 “B” silicon cantilevers (Micromasch, Tallinn, Estonia) with a nominal force constant of 40 N/m. Topographic, phase, and amplitude images at a resolution of 512 × 512 were simultaneously obtained using a scan frequency of 1 Hz with typical scan sizes of 5 *μ*m × 5 μm and 10 *μ*m × 10 *μ*m. Images were processed with a sequence of plane fitting and offset flattening using Gwyddion 2.29 (www.gwyddion.net) software.

### Cell culture

#### Materials

Hank's balanced salt solution (HBSS+), Neurobasal-A, glutamine, penicillin, streptomycin, B27 nutrient mixture, fetal calf serum (FCS), trypsin, and poly-l-ornithine (PLO) were purchased from Invitrogen (Carlsbad, USA). Patch clamp electrode glass was obtained from Harvard Appliances (Kent, UK), the experimental bath was from Warner (Hamden, USA), and glass coverslips were from Menzel-Gläser (Braunschweig, Germany). Primary hippocampal neurons were isolated post-mortem from the brains of E18 Sprague Dawley rat fetuses, using established protocols.^35^

Glass coverslips, with a diameter of 9 mm, were acid-washed, rinsed with 80% ethanol, allowed to dry, and then coated overnight with either PLO control or the self-assembled scaffolds to test their ability to promote neuron adhesion, survival, and function. Next morning, the coating was removed by ×3 washing in HBSS and kept moist until the cells became available (see below).

Primary rat hippocampal neurons were cultured in accordance with standard protocols as previously described.^35^ These procedures were approved by the Monash University Animal Ethics Committee and conform to the Australian National Health and Medical Research Council code of practice for the use of animals in research (MARP/2016/125). In brief, pregnant Sprague Dawley rats were anesthetised on day 18 of pregnancy. The fetuses were removed, and the hippocampi were dissected out. The tissue was washed to remove any remaining red blood cells before being transferred to a solution containing 0.06 mg/ml trypsin in Ca^2+^-free HBSS for 8 min at 37 °C. After incubation, the trypsin solution was removed and the tissues were washed ×3 with HBSS. Hippocampal cells were isolated by gentle trituration with a wide-bore pipette and neuron-containing solution transferred to 5 ml of Dulbecco's minimum medium (DMEM-F12) containing fetal calf serum (FCS) 10%, 50 units/ml of penicillin, and 0.05 mg/ml streptomycin (Pen-strep). This solution was centrifuged at 200*g* for 5 min. One coverslip was placed in each well of a 24-well plate.

Previous work had assumed that fibrous scaffolds were formed within 24 h.^22^ While this protocol was successful, closer examination of self-assembly kinetics was required to optimise fibre formation. Standard 1 mg/ml solutions of **3** or a combination of **3** and **4** (1:3) in water were produced under sterile conditions and placed onto 9 coverslips. The peptides were allowed to self-assemble before the well lids were removed. Removing the lids allows the solvent to evaporate and halts self-assembly. The lid was removed from the wells at 1, 6, and 14 h. Primary rat hippocampal cells were then seeded on the scaffolds. They were maintained at 37 °C, and their growth was monitored for 5 days. Microscopy images were taken daily. Neuron functions were tested using a patch clamp after five days.

The supernatant was aspirated, and the pellet of cells was diluted with DMEM-F12 plus pen-strep such that the cell density was 400,000 cells/ml. The cells were then seeded into the wells of the plate, 0.6 ml of cell solution per well. The plate was placed in the incubator for 4 h, after which time the solution was aspirated and replaced by 1 ml of Neuro-basal A medium containing 20 *μ*l/ml B-27 nutrient additive, 12.5 *μ*l/ml glutamine, and pen-strep. The cells were checked under the microscope and then placed in the incubator. Every 3 days, 700 *μ*l of medium was removed and replaced by 1 ml of fresh medium, and the cells were checked and returned to the incubator. The cells were photographed under a light microscope daily from day 2 or 3 until day 6.

### Electrophysiology

Neuron functions were tested on days 5–7 (inclusive) of culture. The tissue bath was mounted on an inverted microscope, and HBSS, containing (mM) NaCl 137, KCl 5.4, CaCl2 1.5, KH2PO4 0.44, MgCl_2_ 0.5, MgSO_4_ 0.4, Na_2_HPO_4_ 0.3, NaHCO_3_, glucose 5.6, and (4-(2-hydroxyethyl)-1-piperazineethanesulfonic acid) (HEPES) buffer 10, pH 7.4, was continuously flowed through at 1 ml/min and room temperature (∼22 °C). The coverslip on which the cells were growing was gently removed from the culture plate and positioned in the bottom of the tissue bath and left to equilibrate for 10 min. Electrodes were pulled from 1.5 mm glass tubes, and the tip of each electrode was fire-polished. The electrodes were filled with simplified pseudo-cytoplasmic solution containing (in mM) KCl 130, Adenosine triphosphate (ATP) 3, Ethylene-bis(oxyethylenenitrilo)tetraacetic acid (EGTA) 5, MgCl_2_ 1.2, and HEPES buffer 10, pH 7.2. The electrode was mounted on a micromanipulator fixed on the microscope, and square signals, 10 mV in amplitude and 10 ms duration, were obtained at 50 Hz. A cell was selected visually via a camera attached to the microscope, and the electrode was brought up to touch the plasma membrane. The contact between the electrode and the cell membrane was indicated by a reduction in the size of the step responses. A small suction was applied to the electrode, sufficient to rupture the region of the plasma membrane immediately enclosed by the electrode tip (whole cell mode). This created continuity between the contents of the electrode and the cytoplasm. Activity in the neuron was recorded in terms of resting membrane potential (RMP), the occurrence of excitatory post-synaptic potentials (EPSPs) and action potentials (APs), the threshold voltage for the initiation of an AP, the amplitude and half-width of the AP, and whether only a few (tonic) or many (phasic) APs occurred when the neuron was depolarized for 500–1000 ms. The neuron was then voltage clamped, and the sodium current in response to step depolarization (INaV) was established. Data were acquired using an Axoclamp 10 amplifier and analysed using Clampfit software (Axon Instruments, CA, USA). Statistical analysis was conducted with GraphPad using repeated measures of one-way analysis of variance (ANOVA).

### Plasma stability

The stability of peptide **3** or native Ang II (at 1 mg/ml) in rat plasma (diluted 9:1 with saline) incubated at 37 °C was determined as described previously.^36^ Protease activity was quenched in spiked samples by the addition of acetonitrile (water/acetonitrile, 1:1) at selected intervals and stored at −20 °C for analysis. Samples were centrifuged at 10,000*g* for 5 min, and the supernatant was collected for liquid chromatography-mass spectrometry (LC-MS) analysis. The amount of parent compound remaining at each time point was then assayed on an Agilent 1100 MSD SL ion trap mass spectrometer. The peaks observed in the resulting chromatograms were integrated, compared with a standard curve, and cross-checked by mass and retention time.

## SUPPLEMENTARY MATERIAL

See supplementary material for synthetic procedures and data.
